# A Rapid, Simple, Liquid Chromatographic-Electrospray Ionization, Ion Trap Mass Spectrometry Method for the Determination of Finasteride in Human Plasma and its Application to Pharmacokinetic Study 

**Published:** 2012

**Authors:** Syed Husain Hashemi Mousavi, Farzad Kobarfard, Syed Waqif Husain, Mohammad Saber Tehrani, Parviz Abroomand Azar, Reza Ahmadkhaniha, Amir Mehdizadeh

**Affiliations:** a *Department of Chemistry, Science and Research Branch, Islamic Azad University, Tehran, Iran*.; b *Department of Medicinal Chemistry, School of Pharmacy, Phytochemistry Research Center, SBMU, Tehran, Iran.*; c *Pharmacutical Sciences Research Center, Tehran University of Medical Science.*; d *Kimia R&D Institute, Tehran, Iran.*

**Keywords:** Electrospray ionization (ESI), Finasteride; Ion trap, Liquid chromatography–mass spectrometry (LC–MS), Pharmacokinetic

## Abstract

A fast, accurate, sensitive, selective and reliable method using reversed-phase high performance liquid chromatography coupled to electrospray ionization ion trap mass spectrometry was developed and validated for the determination of ﬁnasteride in human plasma. After protein precipitation with perchloric acid, satisfactory separation was achieved on a Zorbax Eclipse^®^ C_8_ analytical column using a mobile phase consisted of acetonitrile, 2 mM ammonium formate buffer (58:42, pH adjusted at 2.5 using formic acid); the flow rate was 0.25 mLmin^-1^ and the column oven was set to 50°C. Tamoxifen citrate was used as internal standard. This method involved the use of [*M *+H]^+ ^ions of ﬁnasteride and IS at *m*/*z *373 and 372 respectively with the selected ion monitoring (SIM) mode. The calibration curve was linear over the range of 0.1–60 ng mL^−1^. The limit of quantiﬁcation for ﬁnasteride in plasma was 0.1 ng mL^−1^. The intra-day and inter-day repeatability (precision) were 2.68-13.87% and 2.14-14.69% respectively. Intra-day and inter-day accuracy were 98-101.57% and 99.7-110%. The assay method has been successfully used to estimate the pharmacokinetics of ﬁnasteride after oral administration of a 5 mg tablet of ﬁnasteride in 12 healthy volunteers.

## Introduction

Finasteride is a synthetic antiandrogen which acts by inhibiting type II 5-alpha reductase, the enzyme that converts testosterone to dihydrotestosterone (DHT). It is used as a treatment in benign prostatic hyperplasia (BPH) in low doses, and prostate cancer in higher doses.

It is also used for treatment of male-pattern baldness (alopecia androgenetica) in men at a dose of 1 mg daily. The study by Thompson *et al*. (1) indicates that Finasteride reduces the rate of prostate cancer by 30%. It is also indicated for use in combination with doxazosin therapy to reduce the risk for symptomatic progression of BPH.

Several methods for determination of finasteride in biological samples have been developed. These methods include high-performance liquid chromatography (HPLC) (2-5), polarography (6), liquid chromatography–tandem mass spectrometry (7-9) and an isotope dilution mass-spectrometric method (10). A review article by Macek discusses these methods in detail (11). HPLC is often the method of choice (13). However, HPLC methods for finasteride measurement in plasma suffer from limitations such as low sensitivity, poor selectivity and being time-consuming due to complex sample preparation procedures. Among these methods, LC–MS/MS method developed by Guo *et al*. (12) may have the highest sensitivity, but the determination process is complex and a two-stage cleaning process and need for using different cartridges seem to be necessary to obtain good selectivity and accuracy. From the reported procedures, the complexity and length of the sample pre-treatment can be reflected by the different limits of quantification (LOQ), which vary from 0.2 to 10 ng mL^−1^. 

**Figure 1:**
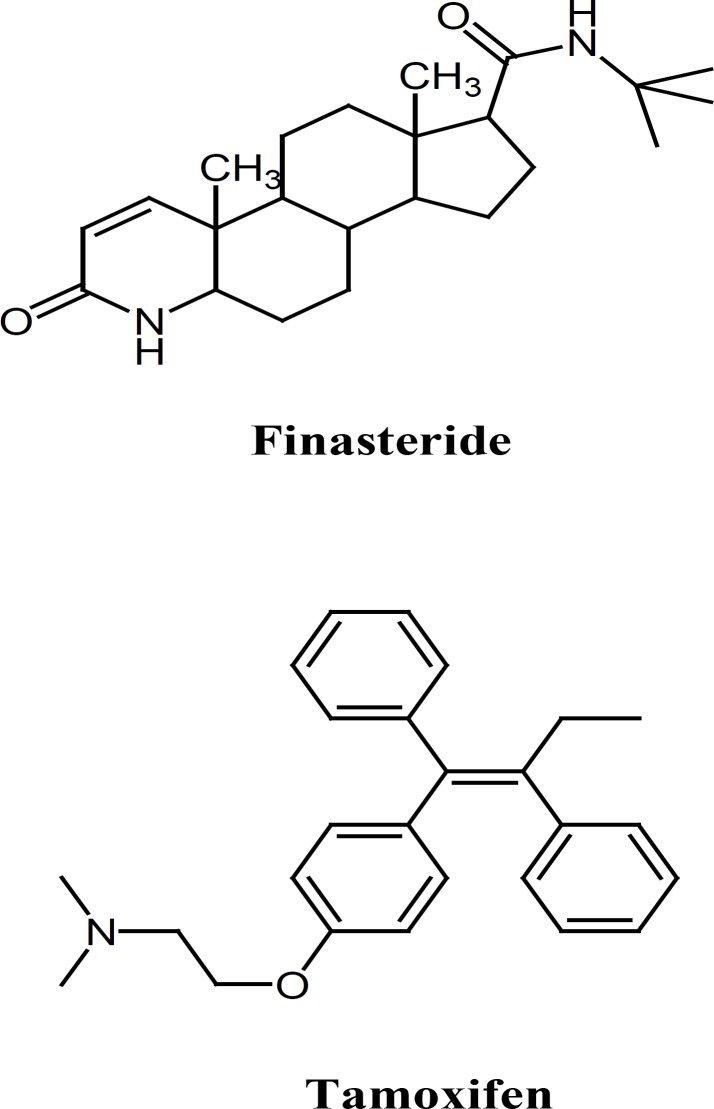
Chemical structures of finasteride and tamoxifen

LC–MS has become the widely used analytical tool for the pharmacokinetic study and quantiﬁcation of drugs and metabolites in biological samples due to its high sensitivity and selectivity. In the present study a fast, sensitive, selective, rapid and accurate liquid chromatographic–electrospray ion trap mass spectrometry (LC/ESI-MS) method for the determination of ﬁnasteride in human plasma is described. The method was validated with good selectivity, linearity range, precision, accuracy, and limit of quantiﬁcation (LOQ). After addition of tamoxifen citrate (Figure 1) as internal standard (IS) and protein precipitation with perchloric acid, a LC–ion trap mass spectrometer with electrospray ionization source (ESI) was used for the quantiﬁcation of ﬁnasteride. By analyzing plasma samples collected from 12 volunteers participating in pharmacokinetic study, the applicability of the developed method was demonstrated.

## Experimental

*Reagents and chemicals*

Reference standards of finasteride and tamoxifen citrate were obtained from Sigma (St. Louis, MO, USA). Oral dosage forms (1 mg tablets) of finasteride were manufactured by Soha Pharmaceutical Co. (Tehran, Iran) and Merck Pharmaceutical Co. HPLC-grade acetonitrile, methanol, ammonium formate, formic acid and perchloric acid were purchased from Merck (Germany). All solvents were ﬁltered through a 0.45 µm membrane and degassed prior to their use in the analyses. Milli-Q grade (Millipore, Bedford, MA, USA) water was used in all cases. Stock solutions of ﬁnasteride and internal standard (IS) tamoxifen citrate at concentration of 0.1 mg mL^−1 ^were prepared in methanol and were stored at −20 ºC. 

*Instrument*

**Figure 2:**
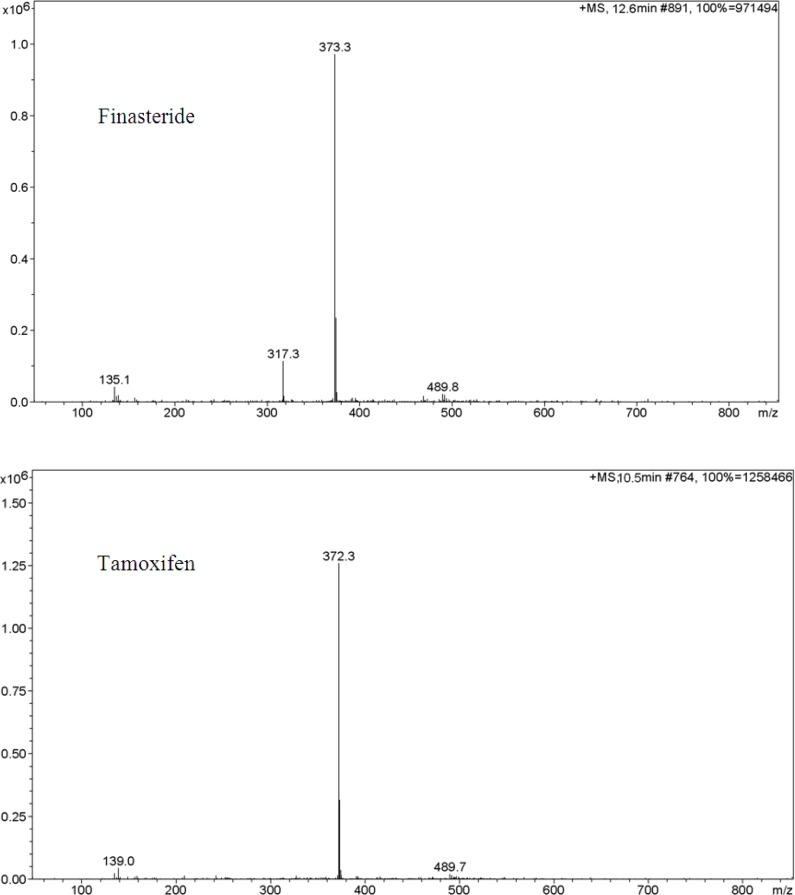
Ion trap LC/ESI-MS mass spectra of finasteride and tamoxifen (IS) in positive scan mode from m/z 100 to 850. Chromatographic conditions and MS parameters are described in Sections 2.3 and 2.4.

An Agilent LC-MS-1100 ion-trap mass spectrometer interfaced with electrospray ionization (ESI) ion source was used. Drying gas temperature was set at 350 ºC. Nebulizing gas flow was 10 Lmin^−1^. Skimmer 1 and skimmer 2 were at 32.1V and 6.0V respectively. Ion charge control (ICC) was on with the target adjusted at 100,000 and maximum accumulation time at 200 ms. Positive selected ion monitoring (SIM) mode and [*M*+H]^+1^ for both finasteride (*m*/*z *373) and tamoxifen (*m*/*z *372) were chosen for determination of finasteride (Figure 2). The data were collected and processed using ChemStation software.

*Chromatographic conditions*

Separations were performed on a 150 mm × 4.6 mm ID, 5 µm particle, Agilent Zorbax Eclipse^®^ C_8_ analytical column. The mobile phase was a mixture of acetonitrile, 2 mM ammonium formate buffer (58:42) and the pH of the mixture was adjusted at 2.5 with formic acid; the flow rate was 0.25 mLmin^-1^ and the column oven was set to 50ºC, total run time was 13 min. The mobile phase was prepared daily.

*Plasma sample preparation*

To a 500 µL aliquot of plasma is added 50 µL tamoxifen solution (1 µg mL^-1^ in methanol) as internal standard and mixed. 100 µL methanol is then added and mixed followed by the addition of 100 µL perchloric acid (70%), mixing for 30 sec and centrifugation at 1048.32 g for 10 min. 50 µL of supernatant is directly injected into the analytical column.

*Assay specificity and matrix effect *

Specificity was assessed by extracting samples of six batches of blank plasma and then comparing the results for plasma samples spiked with tamoxifen (IS) and finasteride. The chromatograms were also inspected visually for interfering chromatographic peak area from endogenous substances.

In order to investigate the effect of ion suppression on mass signals the following procedure was performed: The infusion pump was connected to the HPLC system by a “zero volume tee” before the ion source and the HPLC system pumping the mobile phase, which was the same as that used in the routine analysis of finasteride, *i.e.* acetonitrile: 2 mM ammonium formate buffer (58:42) at 0.25 mLmin^-1^. The infusion pump was set to transfer 30 µLmin^-1^ of a mixture of analyte and internal standard in mobile phase (at 50 ng mL^-1^ and 10 ng mL^-1^ concentration levels for both finasteride and tamoxifen). A sample of human pooled blank plasma was subjected to the sample preparation procedure described in section 2.4. The supernatant was injected into the HPLC system while the standard mixture was being infused. Any ion suppression would be observed as a depression of the MS signal in this system.

*Stability*

The short-term room temperature, long-term storage, stock solution, post-preparative and freeze/thaw stabilities were tested. To test the stability of finasteride in plasma, QC samples were stored under different conditions. The freeze/thaw stability test was performed by freezing-thawing for 3 times. Specifically, freezing was performed at -20 ºC for 24 h and thawed at room temperature. Short-term stability testing was performed at room temperature over 8 h, and long term stability was examined at -20 ºC over 2 months. Post-prepative stability testing was performed by comparing after-day analysis with the first intra-day analysis.

*Validation*

The method was validated for selectivity, linearity, precision, accuracy and recovery. The selectivity test was performed by analyzing the blank plasma sample to test for the interference at the retention time areas of ﬁnasteride and IS. Linearity was tested over the concentration range of 0.1–60.0 ng mL^−1^. In order to construct the calibration curve, a set of eleven non-zero ﬁnasteride calibration standards with concentration of 0.1, 0.25, 0.5, 1.0, 5.0, 10.0, 20.0, 30.0, 40.0, 50.0, 60.0 ng mL^−1^, were prepared by spiking proper amounts of standard solution into blank plasma samples and following the procedure described in section 2.4. The standard samples were treated as described in section 2.4. The retention times of IS and ﬁnasteride were 10.7 and 12.6 min, respectively. The concentrations of unknown samples were calculated using the regression equation of the standard curve. 

Three quality control samples at concentrations of 0.1, 30.0 and 60.0 ng mL^−1^, (LLOQ, MQC and HQC) were prepared by spiking blank plasma samples (using the same stock solutions which were used for the calibration standard), and the added amount of internal standard in each quality control sample was also 100 ng mL^−1^. Sensitivity was determined by analyzing control human plasma samples in replicates (n = 6) spiked with the analyte at the lowest level of the calibration curve, *i.e*. 0.1 ng mL^−1^. Intra-day precision and accuracy were evaluated by analyzing each QC sample six times on the same day, while inter-day precision and accuracy were evaluated by analyzing each QC sample in 6 consecutive days. 

The recovery of ﬁnasteride and IS from plasma samples after addition of perchloric acid was calculated by comparing the peak area response of extracted analytes with unextracted equal amount of standards. Finasteride standard solutions at concentrations of 0.1, 30.0 and 60 ngmL^−1 ^were prepared using mobile phase and standard working solution and the added amount of internal standard was 100 ng mL^−1^. 

*Pharmacokinetic study*

The developed method was used to investigate the plasma concentration–time proﬁle of ﬁnasteride after administration of five 1-mg tablets (total 5 mg) dose. The study was approved by the ethics committee of Shahid Beheshti School of pharmacy. Twelve healthy volunteers (male) participated in the investigation. The age of volunteers was between 24 and 46 years (average 32.4 years). Their body weight was between 55 and 83 kg (mean 71.0 kg) and their body height was 155–178 cm (mean 169.9 cm). All the volunteers took health exam to ensure that they have normal liver, heart rate, blood and electrocardiogram. Before and during the 2 weeks for test, volunteers did not take any other medicine. Following written informed consent, volunteers took five 1-mg tablets of ﬁnasteride with 240 mL tap water. Drinking and smoking were not allowed, and light breakfast was given at 3 h after taking the drug. Lunch was low fat food, given at 5.5 h after taking the drug. Blood samples were collected in heparinized tubes pre-dose (0 h) and at 0.3, 0.6, 1, 1.3, 1.6, 2, 2.3, 2.6, 3, 3.5, 4, 5, 6, 8, 10, and 24 h post-dose. Plasma samples were immediately separated by centrifugation at 143.4 g and stored at −20ºC until analysis. 

## Results and Discussion

*Optimizat* *ion of chromatographic and mass spectrometric conditions*

The developed method was used for a pharmacokinetics study in 12 healthy volunteers. After oral administration of 5 mg finasteride, the concentration versus time proﬁle of ﬁnasteride was constructed. The results showed that the method was reliable and adequate to provide pharmacokinetic concentration–time proﬁle for a dose of finasteride as low as 5 mg. 

The purpose of the present study was to develop a sensitive, simple, fast and reliable LC/ESI-MS method for the determination of ﬁnasteride in human plasma. In the previously published HPLC methods acetonitrile-potassium dihydrogenphosphate water solution or acetonitrile–water (2-5) has been usually used as mobile phase. In some other published methods (12, 11) a drying step after liquid-liquid extraction has been necessary. In the present study, separation of ﬁnasteride and tamoxifen was achieved on an Agilent Zorbax Eclipse^®^ C_8_ analytical column by using a mobile phase consisting of acetonitrile–water. Fnasteride and tamoxifen were protonated in an acidic mobile phase before entering the ionization chamber. 

In order to select an appropriate ionization mode in LC/MS analysis, the mass spectra were acquired in ESI mode by scanning from 50 to 850 amu. The base peak intensities obtained in positive mode were higher than those obtained in negative mode. The positive ion mass spectra of ﬁnasteride and internal standard in scan mode were characterized by a protonated molecular ion [*M *+H]^+ ^at *m*/*z *373 and 372, respectively as base peaks. Therefore, SIM mode involved selective monitoring of *m*/*z *373 and 372 in the vicinity of retention times for finasteride and tamoxifen respectively. The optimized ESI-MS conditions are described in Section 2.2.

**Figure 3:**
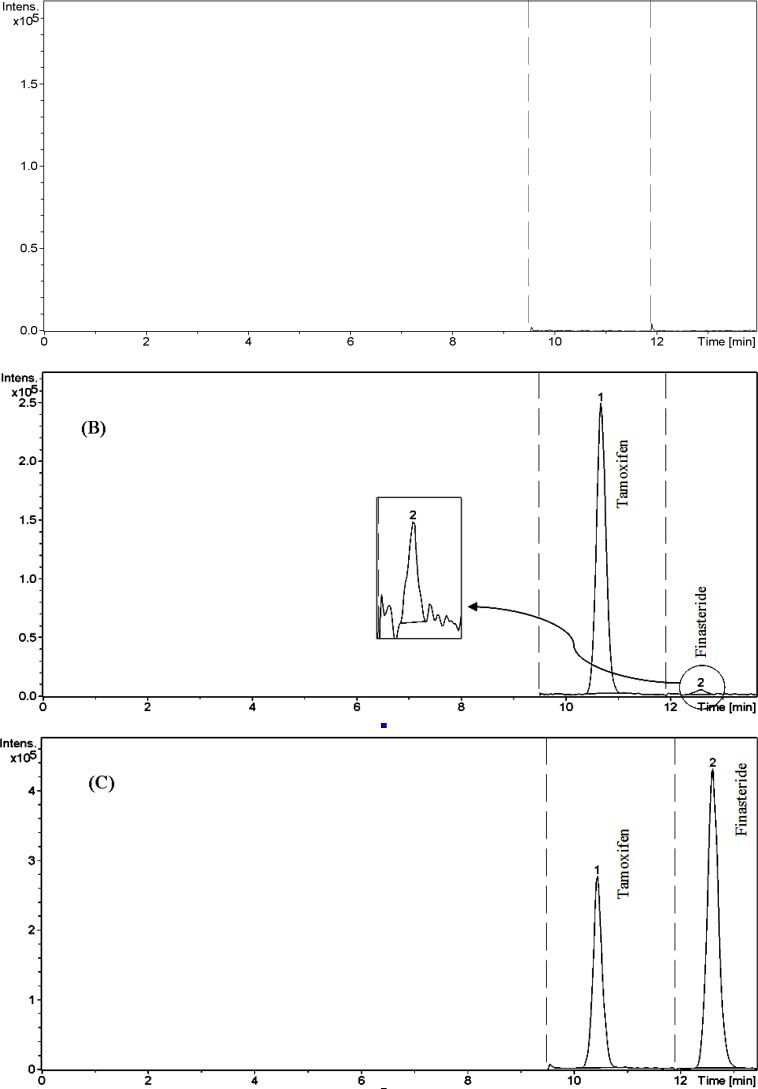
Chromatograms of finasteride and tamoxifen (IS). (A) blank plasma, (B) Plasma spiked with standard (0.1 ng mL −1) and IS (100 ng mL −1), and (C) Plasma spiked with standard (30 ng mL −1) and IS (100 ng mL −1).

A few representative chromatograms of ﬁnasteride and tamoxifen (IS) are shown in Figure 3. The retention times of IS and ﬁnasteride in total ion chromatogram were 10.7 and 12.6 min, respectively.

*Selectivity* *and matrix effect*

The LC/ESI–MS method shows high selectivity because only selected ions from the analytes of interest are monitored. Chromatograms of the blank and the spiked human plasma samples (see Figure 3.) indicated no signiﬁcant interferences at the retention time areas of the analyte and IS. ESI positive MS spectra for finasteride and tamoxifen were dominated by the [M+1]^+ ^ions, *i.e.* *m*/*z* 373 for finasteride and 372 for tamoxifen. 

It was very important to investigate the matrix effects to develop a reliable and reproducible LC/ESI–MS method. Here, the matrix effect was evaluated by the following experiments: finasteride and tamoxifen were spiked separately into human blank plasma as well as into the mobile phase as solvent.

After being treated according to the procedure described in Section 2.4, these samples were injected into LC/ESI-MS. No significant difference was observed between the peak area in chromatogram of spiked plasma samples and the peak area in the chromatogram obtained by injection of the solution of finasteride and IS in mobile phase. It was also shown that no endogenous compounds signiﬁcantly inﬂuenced the ionization of ﬁnasteride and IS. Furthermore, in the set up described in section 2.5 no significant ion suppression was observed for the MS signals of finasteride and tamoxifen.

*Linearity*

The linear regression equation, was *y *= 0.102*x* + 0.022 with correlation coefficient being 0.999 which its y and x are the ratio of peak area of finasteride per IS and finasteride concentration, respectively. Other linear regression data are SD = 0.0448, slop = 0.102 and intercept = 0.022. The method reported here is very sensitive due to using optimum ESI-MS conditions and the advantages of LC/ESI-MS in the selected ion monitoring (SIM) mode. The lowest standard concentration in the calibration curve was considered as the lower limit of quantiﬁcation (LLOQ), which was 0.1 ng mL^−1^. For LLOQ, the mean deviation percentage from the nominal concentration was 8.5% and precision was 13.9%. A good signal-to-noise ratio (10:1) was observed at the LLOQ indicating that the corresponding value could be reached. The sensitivity of the developed method was higher than previously published HPLC methods (2-5) and polarography (6) for determination of finasteride in plasma.

**Table 1:** Intra-day precision and accuracy of quality control samples for the determination of finasteride in human plasma

**Added concentration (ng mL** ^−1^ **)**	**Mean found concentration ** **(ng mL** ^−1^ **)**	**accuracy %**	**precision % (R.S.D.)**
0.1	0.098	98.00	13.87
30	30.47	101.57	5.75
60	59.71	99.52	2.68

The sensitivity of the developed method was also comparable with that of the previously reported LC/MS-MS methods (7, 9, 11, 12). The lowest LLOQ reported for LC/MS-MS is 0.2 ng mL^−1^ which is comparable with the LLOQ of our method (0.1 ng mL^−1^). However the reported dynamic range for LC/MS-MS is 0.2-120.0 ng ml^−1^ which is wider than the dynamic range of our method (0.1–60.0 ng mL^−1^). This is due to the nature of ion trap mass analyzer which has a limited space for ion accommodation. In this analyzer, accumulation of ions higher that a certain amount in the trap leads to the cross talk among them which adversely affect the linearity of the responses produced by the system. Furthermore, the sample preparation procedure which consists of one-step protein precipitation and direct injection is much simpler than those of other LC-MS methods (12) comprising the use of SPE and liquid–liquid extraction.

*Precision, accuracy and recovery*

Both the intra-and inter-day accuracy and precision of the developed method were determined by six replicate analyses of quality control samples containing known concentrations of ﬁnasteride ranging from 0.1 to 60.0 ng mL^−1^. The precision of the method was described as relative standard deviation (R.S.D.). The accuracy was described as a percentage of measured concentrations versus nominal concentrations of ﬁnasteride in QC samples. The results of intra-and inter-day accuracy and precision are listed in Table 1 and 2. 

Composition of the mobile phase was found to be the critical factor for achieving good chromatographic peak shape and resolution. In the present study, a mixture of 2.5 mmol/L ammonium acetate solution and acetonitrile, (42:58 v/v, pH adjusted at 2.6) was used as a mobile phase. The selection of tamoxifen as the internal standard was based on our previous experience on ion-trap LC-MS system which gives robust and reproducible signal for this compound.

The matrix effect was minimal and no co-eluting endogenous compound interfered with the ionization of the analyte and internal standard. This was mostly due to the low pH of mobile phase (2.^6^) and the use of perchloric acid for protein precipitation which minimizes the ion suppression. 

**Table 2:** Inter-day precision and accuracy of quality control samples for the determination of finasteride in human plasma

**Added concentration (ng mL** ^−1^ **)**	**Mean found concentration (ng mL** ^−1^ **)**	**accuracy %**	**precision % (R.S.D.)**
0.1	0.11	110	14.69
30	30.55	101.83	4.32
60	59.89	99.77	2.14

**Figure 4:**
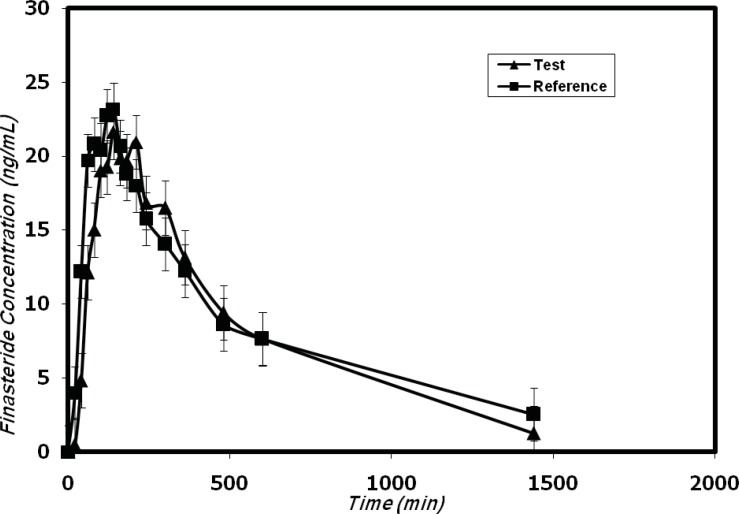
Mean plasma concentrations profiles of finasteride in 12 healthy volunteers after oral administration of the test (trademark: Finasteride Soha) and reference (trademark: Propecia).

The lower limit of quantification (LLOQ) is two fold better than the lowest quantification limit reported by Guo *et al*. (12), on a quadropole LC-MS system. This could be due to the cumulative nature of ion trap mass analyzer in comparison to quadrupole analyzer in which ions are quickly pass the mass analyzer and do not get the chance of accumulation. Further more, the method reported by Guo *et al*. (12) involves sample extraction procedures and tandem mass spectrometry, two parameters which are not implicated in the present method.

*Stability*

Stability was evaluated as a part of method validation. Finasteride standard at concentrations of 0.1, 30.0 and 60.0 ng mL^−1 ^(LLQC, MQC and HQC) were used for stability experiments. Ten milliliters of 0.1, 30.0 and 60.0 ng mL^−1 ^ﬁnasteride standard solutions were prepared by diluting standard solution with blank plasma, and the added amount of internal standard in each quality control sample was 100 ng mL^−1^. The results indicated that the difference of measured concentration from time 0 to 8 h was less than 4.1% when these samples were placed at room temperature, which allowed us to conclude that processed samples were stable for at least 8 h. When these standard solutions were stored at −20ºC, four thaw and freeze cycles were performed before being processed as described in section 2.4. During each cycle, 1 mL standard was processed. The difference of measured concentration from nominal concentration was less than 5.0% for 0.2, 30.0 and 60.0 ng mL^−1^, and the results indicated that the stability of ﬁnasteride was not affected by freezing and thawing. In long-term stability experiments, after storage for 1 month at −20°C, more than 96.1% of ﬁnasteride remained according to their peak areas at each concentration. 

*Application*

The developed method was successfully used for the determination of plasma concentrations of ﬁnasteride after oral administration of five 1-mg tablets (total 5 mg) dose to 12 healthy volunteers in a bioequivalence study for a generic formulation of finasteride (trademark: Finasteride Soha) and the reference formulation (trademark: Propecia). Figure 4 shows the finasteride plasma mean concentration vs. time profile obtained after the single oral administration of 5 mg of Finasteride Soha (test) and Propecia (reference) in 12 volunteers.

The pharmacokinetic parameters are shown in Table 3. The obtained values are consistent with previously published report (12, 14) which indicated the suitability of the developed analytical method for pharmacokinetic studies.

## Conclusions

A fast, sensitive, specific LC/ESI-MS method for the determination of finasteride in human plasma was developed and validated. Compared with the previously published methods, significantly lower limit of quantification, which is 0.1 ng mL-1, was obtained. Using of 0.5 mL aliquot of plasma sample and protein precipitation with perchloric acid, are the advantages of this method. The method has been successfully applied to the pharmacokinetics studies and satisfactory results were obtained, which demonstrates that the method is reproducible, sensitive and reliable.
